# Crystal structure of ethyl 3-(4-chloro­phen­yl)-5-[(*E*)-2-(di­methyl­amino)­ethen­yl]-1,2-oxazole-4-carboxyl­ate

**DOI:** 10.1107/S2056989015023257

**Published:** 2015-12-09

**Authors:** Ilya Efimov, Pavel Slepukhin, Vasiliy Bakulev

**Affiliations:** a Ural Federal University, Mira 19 Ekaterinburg 620002, Russian Federation; b I. Postovsky Institute of Organic Synthesis, Kovalevskoy 22 Ekaterinburg 620090, Russian Federation

**Keywords:** crystal structure, enamine, isoxazole

## Abstract

In the title compound, C_16_H_17_ClN_2_O_3_, two mol­ecules, *A* and *B*, with different conformations, comprise the asymmetric unit. In mol­ecule *A*, the C=O group of the ester points away from the benzene ring [C—C—C=O = −170.8 (3)°], whereas in mol­ecule *B*, it points back towards the benzene ring [C—C—C=O = 17.9 (4)°]. The dihedral angles betweeen the oxazole and benzene rings also differ somewhat [46.26 (13) for mol­ecule *A* and 41.59 (13) for mol­ecule *B*]. Each mol­ecule features an intra­molecular C—H⋯O inter­action, which closes an *S*(6) ring. In the crystal, the *B* mol­ecules are linked into [001] *C*(12) chains by weak C—H⋯Cl inter­actions.

## Related literature   

For related literature, see: Bakulev *et al.* (2012 ▸, 2013 ▸); Bredereck *et al.* (1967 ▸).
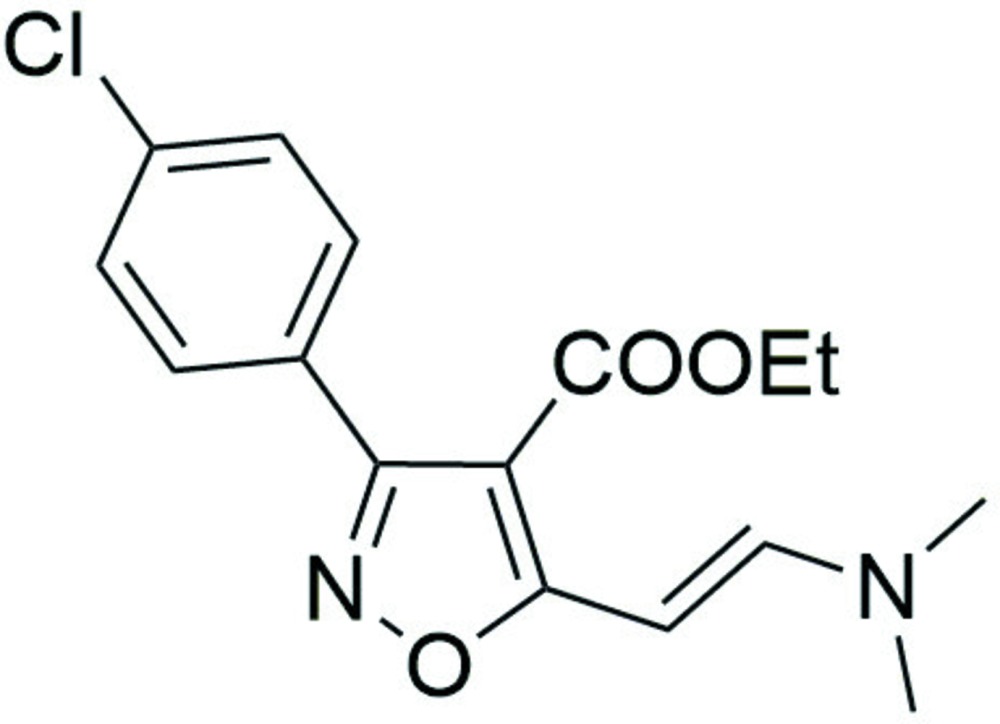


## Experimental   

### Crystal data  

C_16_H_17_ClN_2_O_3_
*M* *_r_* = 320.77
Triclinic, 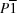

*a* = 11.5494 (13) Å
*b* = 12.474 (2) Å
*c* = 13.0430 (13) Å
α = 102.369 (11)°
β = 105.501 (9)°
γ = 109.001 (13)°
*V* = 1615.8 (4) Å^3^
*Z* = 4
Mo *K*α radiation
μ = 0.25 mm^−1^
*T* = 295 K
0.25 × 0.20 × 0.15 mm

### Data collection  

Oxford Diffraction Xcalibur S CCD diffractometer
Absorption correction: multi-scan (*CrysAlis RED*; Oxford Diffraction, 2006 ▸) *T*_min_ = 0.061, *T*_max_ = 0.962
14310 measured reflections
6536 independent reflections
2382 reflections with *I* > 2σ(*I*)
*R*_int_ = 0.031

### Refinement  

*R*[*F* ^2^ > 2σ(*F* ^2^)] = 0.039
*wR*(*F* ^2^) = 0.082
*S* = 1.00
6536 reflections
414 parameters
H atoms treated by a mixture of independent and constrained refinement
Δρ_max_ = 0.22 e Å^−3^
Δρ_min_ = −0.19 e Å^−3^

### 

Data collection: *CrysAlis CCD* (Oxford Diffraction, 2006 ▸); cell refinement: *CrysAlis RED* (Oxford Diffraction, 2006 ▸); data reduction: *CrysAlis RED*; program(s) used to solve structure: *SHELXS97* (Sheldrick, 2008 ▸); program(s) used to refine structure: *SHELXL97* (Sheldrick, 2008 ▸); molecular graphics: *SHELXTL* (Sheldrick, 2008 ▸); software used to prepare material for publication: *SHELXTL*.

## Supplementary Material

Crystal structure: contains datablock(s) I. DOI: 10.1107/S2056989015023257/hb7538sup1.cif

Structure factors: contains datablock(s) I. DOI: 10.1107/S2056989015023257/hb7538Isup2.hkl

Supporting information file. DOI: 10.1107/S2056989015023257/hb7538Isup3.cdx

Supporting information file. DOI: 10.1107/S2056989015023257/hb7538Isup5.cdx

Supporting information file. DOI: 10.1107/S2056989015023257/hb7538Isup5.cml

**Fig. 1.**
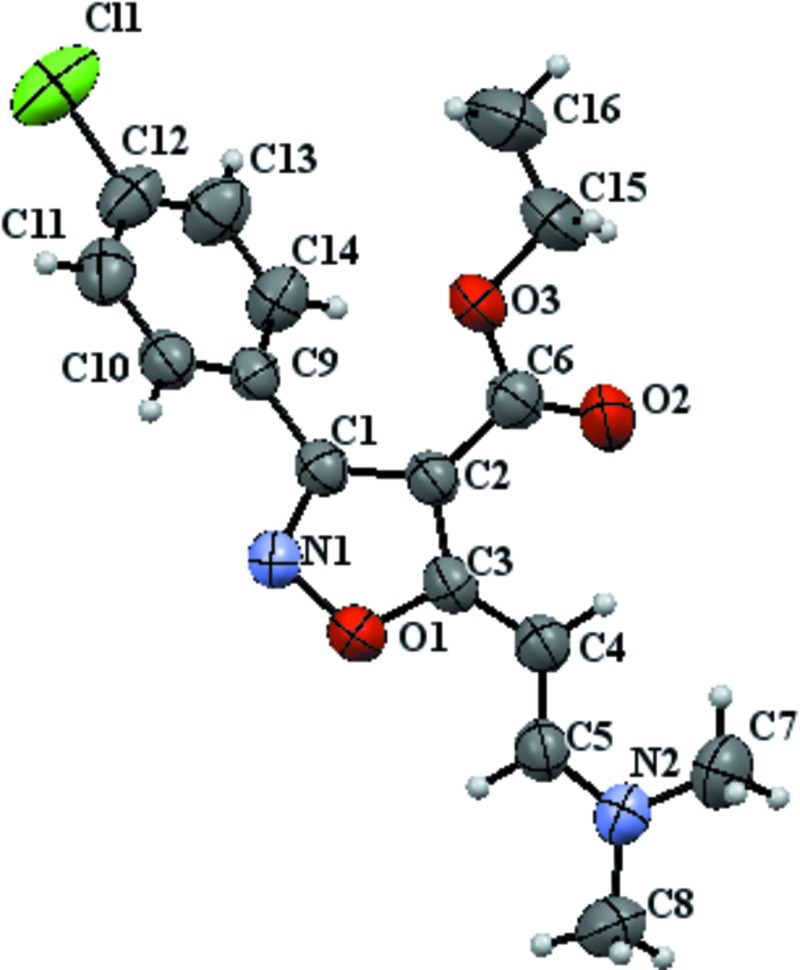
Ellipsoid plot

. DOI: 10.1107/S2056989015023257/hb7538fig1.tif
Ellipsoid plot

CCDC reference: 1440290

Additional supporting information:  crystallographic information; 3D view; checkCIF report

## Figures and Tables

**Table 1:** Hydrogen-bond geometry (Å, °)

*D*—H⋯*A*	*D*—H	H⋯*A*	*D*⋯*A*	*D*—H⋯*A*
C4—H4⋯O2	0.95 (2)	2.425 (18)	3.025 (3)	121.1 (13)
C4*A*—H4*A*⋯O3*A*	0.91 (2)	2.409 (19)	2.996 (3)	122.7 (14)
C16*A*—H16*F*⋯Cl1*A* ^i^	0.96	2.74	3.607 (3)	151

Symmetry code: (i) 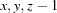.

## supplementary crystallographic information

### S1. Chemical context

This enamine was synthesized for the reactions [3+2] cyclo­addition with azides [3] and hydroxamoylchlorides [4].

### S2. Structural commentary

In according XRD data, two independent molecules are cristallised in the centrosymmetric unit cell. The independent molecules are distinguishes in the orientation of the COOEt-groups and some insignificant details; the general view of the molecules and numeration of the atoms are presented on the fig.1. The bonds lengths and angles in both molecules are in good agreement with standards. The 4-ClPh-substituents are turned toward the heterocyclic planes on the angle 41 and 46o. The non-hydrogen atoms of the =C-NMe2 groups are placed in the plane in the limits 0.04 Å. The bond lengths in the enamine moieties (N(2)—C(5)= 1.336 (3), N(2A)—C(5A)= 1.326 (2), C(4)—C(5)= 1.355 (3), C(4A)—C(5A)= 1.354 (3)Å) show a strong conjugation in the N—C=C group. No any shortened inter­molecular contacts in the crystal are observed.

### S3. Synthesis and crystallization

The Bredereck reagent [1] (0.553 g, 3 mmol) was added to ethyl 3-(4-chloro­phenyl)-5-methyl-1,2-oxazole-4-carboxyl­ate (0.266 g, 1 mmol) and heated for 2 h at 50°C. The reaction product was cooled and hexane was added. The precipitate was filtered off, dried, and recrystallized from EtOH.

### S4. Refinement

Crystal data, data collection and structure refinement details are summarized in Table 1.

### Crystal data

**Table:**

C_16_H_17_ClN_2_O_3_	*Z* = 4
*M**_r_* = 320.77	*F*(000) = 672
Triclinic, *P*1	*D*_x_ = 1.319 Mg m^−^^3^
*a* = 11.5494 (13) Å	Mo *K*α radiation, λ = 0.71073 Å
*b* = 12.474 (2) Å	Cell parameters from 2382 reflections
*c* = 13.0430 (13) Å	θ = 2.8–26.4°
α = 102.369 (11)°	µ = 0.25 mm^−^^1^
β = 105.501 (9)°	*T* = 295 K
γ = 109.001 (13)°	Prism, colorless
*V* = 1615.8 (4) Å^3^	0.25 × 0.20 × 0.15 mm

### Data collection

**Table:**

Oxford Diffraction Xcalibur S CCD diffractometer	6536 independent reflections
Radiation source: fine-focus sealed tube	2382 reflections with *I* > 2σ(*I*)
Graphite monochromator	*R*_int_ = 0.031
ω scans	θ_max_ = 26.4°, θ_min_ = 2.8°
Absorption correction: multi-scan (*CrysAlis RED*; Oxford Diffraction, 2006)	*h* = −13→14
*T*_min_ = 0.061, *T*_max_ = 0.962	*k* = −15→15
14310 measured reflections	*l* = −16→16

### Refinement

**Table:**

Refinement on *F*^2^	Secondary atom site location: difference Fourier map
Least-squares matrix: full	Hydrogen site location: inferred from neighbouring sites
*R*[*F*^2^ > 2σ(*F*^2^)] = 0.039	H atoms treated by a mixture of independent and constrained refinement
*wR*(*F*^2^) = 0.082	*w* = 1/[σ^2^(*F*_o_^2^) + (0.0245*P*)^2^] where *P* = (*F*_o_^2^ + 2*F*_c_^2^)/3
*S* = 1.00	(Δ/σ)_max_ = 0.001
6536 reflections	Δρ_max_ = 0.22 e Å^−^^3^
414 parameters	Δρ_min_ = −0.19 e Å^−^^3^
0 restraints	Extinction correction: *SHELXTL* (Sheldrick, 2008), Fc^*^=kFc[1+0.001xFc^2^λ^3^/sin(2θ)]^-1/4^
Primary atom site location: structure-invariant direct methods	Extinction coefficient: 0.0196 (8)

### Special details

**Table:**

Experimental. Absorption correction: CrysAlis RED, Oxford Diffraction Ltd., Version 1.171.29.9 (release 23-03-2006 CrysAlis171 .NET) (compiled Mar 23 2006,23:39:28) Empirical absorption correction using spherical harmonics, implemented in SCALE3 ABSPACK scaling algorithm.
Geometry. All e.s.d.'s (except the e.s.d. in the dihedral angle between two l.s. planes) are estimated using the full covariance matrix. The cell e.s.d.'s are taken into account individually in the estimation of e.s.d.'s in distances, angles and torsion angles; correlations between e.s.d.'s in cell parameters are only used when they are defined by crystal symmetry. An approximate (isotropic) treatment of cell e.s.d.'s is used for estimating e.s.d.'s involving l.s. planes.
Refinement. Refinement of *F*^2^ against ALL reflections. The weighted *R*-factor *wR* and goodness of fit *S* are based on *F*^2^, conventional *R*-factors *R* are based on *F*, with *F* set to zero for negative *F*^2^. The threshold expression of *F*^2^ > σ(*F*^2^) is used only for calculating *R*-factors(gt) *etc*. and is not relevant to the choice of reflections for refinement. *R*-factors based on *F*^2^ are statistically about twice as large as those based on *F*, and *R*- factors based on ALL data will be even larger.

### Fractional atomic coordinates and isotropic or equivalent isotropic displacement parameters (Å^2^)

**Table:**

	*x*	*y*	*z*	*U*_iso_*/*U*_eq_	
Cl1	0.72042 (8)	0.13874 (7)	0.46885 (6)	0.1170 (3)	
O1	1.11936 (13)	0.43268 (13)	1.13549 (13)	0.0683 (5)	
C1	0.9661 (2)	0.32652 (19)	0.97111 (19)	0.0534 (6)	
N1	1.08897 (17)	0.40459 (16)	1.01763 (16)	0.0664 (6)	
N2	1.15752 (18)	0.47264 (17)	1.46652 (17)	0.0671 (6)	
O2	0.74356 (14)	0.21389 (15)	1.11743 (14)	0.0820 (5)	
C2	0.9124 (2)	0.30189 (19)	1.05367 (19)	0.0538 (6)	
O3	0.71582 (14)	0.14117 (14)	0.93699 (14)	0.0819 (5)	
C3	1.0127 (2)	0.3703 (2)	1.1554 (2)	0.0566 (6)	
C4	1.0238 (2)	0.3823 (2)	1.2679 (2)	0.0605 (7)	
C5	1.1332 (3)	0.4606 (2)	1.3577 (2)	0.0623 (7)	
C6	0.7845 (2)	0.2175 (2)	1.0421 (2)	0.0621 (7)	
C7	1.0702 (2)	0.3862 (2)	1.49972 (19)	0.0896 (8)	
H7A	0.9939	0.3316	1.4347	0.134*	
H7B	1.0436	0.4275	1.5539	0.134*	
H7C	1.1150	0.3421	1.5326	0.134*	
C8	1.2785 (2)	0.5627 (2)	1.55382 (18)	0.0862 (8)	
H8A	1.3275	0.6144	1.5213	0.129*	
H8B	1.3299	0.5242	1.5883	0.129*	
H8C	1.2585	0.6095	1.6098	0.129*	
C9	0.9064 (2)	0.28156 (18)	0.84677 (19)	0.0505 (6)	
C10	0.9715 (2)	0.24439 (19)	0.7822 (2)	0.0619 (7)	
H10A	1.0549	0.2483	0.8175	0.074*	
C11	0.9155 (3)	0.20136 (19)	0.6659 (2)	0.0704 (7)	
H11A	0.9606	0.1763	0.6232	0.084*	
C12	0.7931 (3)	0.1960 (2)	0.6141 (2)	0.0695 (7)	
C13	0.7275 (2)	0.2348 (2)	0.6764 (2)	0.0775 (8)	
H13A	0.6449	0.2325	0.6408	0.093*	
C14	0.7843 (2)	0.2773 (2)	0.7921 (2)	0.0685 (7)	
H14A	0.7394	0.3036	0.8343	0.082*	
C15	0.5864 (2)	0.0541 (3)	0.9179 (2)	0.1129 (11)	
H15A	0.5951	−0.0082	0.9489	0.135*	
H15B	0.5424	0.0934	0.9563	0.135*	
C16	0.5094 (2)	0.0015 (3)	0.8002 (2)	0.1273 (12)	
H16A	0.4242	−0.0558	0.7891	0.191*	
H16B	0.5524	−0.0383	0.7625	0.191*	
H16C	0.4999	0.0631	0.7699	0.191*	
Cl1A	1.83045 (7)	0.87414 (7)	1.92457 (5)	0.1147 (3)	
O1A	1.44539 (12)	0.59724 (12)	1.25653 (12)	0.0600 (4)	
N1A	1.47772 (16)	0.61535 (15)	1.37384 (15)	0.0564 (5)	
C1A	1.59219 (19)	0.70750 (18)	1.42308 (18)	0.0472 (6)	
N2A	1.39346 (16)	0.58880 (16)	0.93108 (16)	0.0622 (5)	
O2A	1.85742 (15)	0.88133 (16)	1.45308 (14)	0.1024 (7)	
C2A	1.63778 (18)	0.75251 (18)	1.34370 (18)	0.0468 (6)	
O3A	1.76896 (12)	0.89249 (13)	1.28499 (12)	0.0678 (5)	
C3A	1.5409 (2)	0.68132 (19)	1.23979 (19)	0.0497 (6)	
C4A	1.5256 (2)	0.6768 (2)	1.1277 (2)	0.0546 (7)	
C5A	1.4154 (2)	0.5992 (2)	1.0386 (2)	0.0531 (6)	
C6A	1.7652 (2)	0.8482 (2)	1.3683 (2)	0.0596 (7)	
C7A	1.4919 (2)	0.6602 (2)	0.89544 (18)	0.0842 (8)	
H7AA	1.5700	0.7116	0.9601	0.126*	
H7AB	1.4585	0.7083	0.8581	0.126*	
H7AC	1.5124	0.6080	0.8445	0.126*	
C8A	1.2676 (2)	0.5056 (2)	0.84385 (17)	0.0785 (8)	
H8AA	1.2109	0.4642	0.8776	0.118*	
H8AB	1.2809	0.4484	0.7913	0.118*	
H8AC	1.2278	0.5493	0.8050	0.118*	
C9A	1.65053 (17)	0.7493 (2)	1.54694 (19)	0.0466 (6)	
C10A	1.64545 (18)	0.6682 (2)	1.60521 (19)	0.0541 (6)	
H10B	1.6045	0.5863	1.5650	0.065*	
C11A	1.69924 (19)	0.7054 (2)	1.7205 (2)	0.0601 (6)	
H11B	1.6956	0.6495	1.7581	0.072*	
C12A	1.7583 (2)	0.8255 (3)	1.77963 (19)	0.0668 (7)	
C13A	1.7626 (2)	0.9079 (2)	1.7248 (2)	0.0753 (8)	
H13B	1.8018	0.9895	1.7660	0.090*	
C14A	1.7091 (2)	0.8706 (2)	1.6091 (2)	0.0656 (7)	
H14B	1.7122	0.9271	1.5724	0.079*	
C15A	1.8889 (2)	0.9926 (2)	1.3032 (2)	0.0817 (8)	
H15C	1.9070	1.0590	1.3683	0.098*	
H15D	1.9620	0.9687	1.3184	0.098*	
C16A	1.8769 (2)	1.0293 (3)	1.2094 (2)	0.1289 (13)	
H16D	1.9571	1.0953	1.2227	0.193*	
H16E	1.8056	1.0544	1.1953	0.193*	
H16F	1.8597	0.9638	1.1452	0.193*	
H5	1.2040 (17)	0.5145 (15)	1.3454 (14)	0.052 (6)*	
H4	0.9500 (16)	0.3281 (16)	1.2759 (14)	0.052 (6)*	
H5A	1.3451 (17)	0.5437 (16)	1.0551 (14)	0.060 (7)*	
H4A	1.5937 (18)	0.7341 (17)	1.1224 (15)	0.066 (7)*	

### Atomic displacement parameters (Å^2^)

**Table:**

	*U*^11^	*U*^22^	*U*^33^	*U*^12^	*U*^13^	*U*^23^
Cl1	0.1601 (7)	0.1288 (7)	0.0607 (5)	0.0742 (6)	0.0246 (5)	0.0239 (5)
O1	0.0583 (10)	0.0680 (11)	0.0602 (12)	0.0077 (8)	0.0204 (8)	0.0163 (9)
C1	0.0518 (15)	0.0481 (15)	0.0580 (17)	0.0164 (12)	0.0209 (13)	0.0183 (13)
N1	0.0643 (13)	0.0693 (14)	0.0542 (14)	0.0123 (11)	0.0247 (11)	0.0182 (11)
N2	0.0685 (13)	0.0777 (15)	0.0547 (15)	0.0253 (12)	0.0254 (12)	0.0242 (12)
O2	0.0667 (10)	0.1044 (14)	0.0722 (13)	0.0197 (9)	0.0326 (10)	0.0387 (11)
C2	0.0469 (14)	0.0549 (15)	0.0576 (17)	0.0158 (12)	0.0202 (13)	0.0213 (13)
O3	0.0632 (11)	0.0821 (12)	0.0689 (13)	−0.0058 (9)	0.0258 (9)	0.0184 (10)
C3	0.0523 (15)	0.0561 (16)	0.0629 (19)	0.0184 (13)	0.0248 (14)	0.0234 (14)
C4	0.0539 (17)	0.0658 (18)	0.060 (2)	0.0205 (15)	0.0216 (15)	0.0236 (15)
C5	0.0599 (18)	0.0672 (18)	0.065 (2)	0.0244 (15)	0.0299 (17)	0.0254 (16)
C6	0.0615 (17)	0.0660 (18)	0.0600 (19)	0.0247 (14)	0.0199 (15)	0.0280 (16)
C7	0.0998 (19)	0.102 (2)	0.0706 (19)	0.0342 (17)	0.0354 (15)	0.0416 (17)
C8	0.0790 (17)	0.097 (2)	0.0600 (18)	0.0254 (16)	0.0157 (15)	0.0114 (16)
C9	0.0529 (14)	0.0430 (14)	0.0531 (17)	0.0133 (12)	0.0206 (14)	0.0193 (12)
C10	0.0577 (14)	0.0579 (16)	0.0676 (19)	0.0184 (12)	0.0274 (15)	0.0180 (14)
C11	0.0856 (19)	0.0583 (17)	0.073 (2)	0.0274 (15)	0.0407 (16)	0.0212 (15)
C12	0.094 (2)	0.0647 (18)	0.0508 (18)	0.0356 (16)	0.0231 (17)	0.0198 (14)
C13	0.0839 (18)	0.091 (2)	0.065 (2)	0.0468 (16)	0.0217 (17)	0.0311 (16)
C14	0.0795 (18)	0.0774 (19)	0.0585 (19)	0.0393 (15)	0.0292 (14)	0.0247 (15)
C15	0.0746 (18)	0.114 (2)	0.096 (2)	−0.0194 (17)	0.0269 (17)	0.0275 (19)
C16	0.0691 (18)	0.143 (3)	0.108 (3)	0.0006 (18)	0.0206 (18)	0.006 (2)
Cl1A	0.1036 (5)	0.1297 (7)	0.0551 (5)	0.0072 (5)	0.0147 (4)	0.0065 (4)
O1A	0.0552 (9)	0.0556 (10)	0.0515 (11)	0.0063 (8)	0.0134 (8)	0.0172 (8)
N1A	0.0523 (11)	0.0566 (13)	0.0516 (13)	0.0124 (10)	0.0153 (10)	0.0213 (10)
C1A	0.0426 (13)	0.0433 (15)	0.0505 (16)	0.0149 (12)	0.0121 (12)	0.0158 (13)
N2A	0.0564 (12)	0.0686 (14)	0.0502 (14)	0.0122 (10)	0.0199 (11)	0.0179 (11)
O2A	0.0508 (10)	0.1411 (17)	0.0742 (13)	−0.0074 (10)	0.0020 (9)	0.0571 (12)
C2A	0.0415 (13)	0.0462 (14)	0.0462 (15)	0.0127 (11)	0.0126 (12)	0.0151 (12)
O3A	0.0527 (9)	0.0668 (11)	0.0606 (11)	−0.0018 (8)	0.0124 (8)	0.0284 (9)
C3A	0.0462 (14)	0.0461 (15)	0.0595 (18)	0.0161 (12)	0.0231 (13)	0.0220 (14)
C4A	0.0456 (15)	0.0557 (17)	0.0537 (18)	0.0115 (13)	0.0158 (14)	0.0182 (14)
C5A	0.0543 (16)	0.0553 (17)	0.0500 (18)	0.0183 (13)	0.0226 (14)	0.0194 (14)
C6A	0.0496 (16)	0.0631 (17)	0.0583 (18)	0.0126 (14)	0.0173 (13)	0.0246 (15)
C7A	0.0800 (16)	0.097 (2)	0.0693 (19)	0.0178 (15)	0.0386 (15)	0.0298 (16)
C8A	0.0668 (16)	0.0863 (19)	0.0514 (16)	0.0128 (14)	0.0114 (13)	0.0061 (14)
C9A	0.0402 (12)	0.0425 (16)	0.0532 (16)	0.0111 (11)	0.0178 (11)	0.0165 (13)
C10A	0.0475 (13)	0.0472 (15)	0.0582 (17)	0.0120 (11)	0.0154 (12)	0.0158 (14)
C11A	0.0563 (14)	0.0627 (18)	0.0554 (18)	0.0177 (13)	0.0176 (13)	0.0223 (14)
C12A	0.0553 (15)	0.078 (2)	0.0474 (17)	0.0095 (14)	0.0173 (13)	0.0137 (16)
C13A	0.0778 (17)	0.0539 (18)	0.066 (2)	0.0036 (14)	0.0283 (15)	0.0002 (16)
C14A	0.0764 (16)	0.0468 (17)	0.0667 (19)	0.0147 (13)	0.0289 (14)	0.0185 (14)
C15A	0.0531 (15)	0.0800 (19)	0.089 (2)	−0.0045 (14)	0.0247 (14)	0.0343 (16)
C16A	0.099 (2)	0.143 (3)	0.115 (3)	0.0002 (19)	0.0226 (18)	0.086 (2)

### Geometric parameters (Å, º)

**Table:**

Cl1—C12	1.728 (2)	Cl1A—C12A	1.724 (2)
O1—C3	1.351 (2)	O1A—C3A	1.356 (2)
O1—N1	1.4177 (19)	O1A—N1A	1.4224 (18)
C1—N1	1.310 (2)	N1A—C1A	1.312 (2)
C1—C2	1.419 (3)	C1A—C2A	1.419 (3)
C1—C9	1.477 (3)	C1A—C9A	1.471 (3)
N2—C5	1.336 (3)	N2A—C5A	1.326 (2)
N2—C8	1.446 (2)	N2A—C7A	1.450 (2)
N2—C7	1.447 (3)	N2A—C8A	1.453 (2)
O2—C6	1.202 (2)	O2A—C6A	1.190 (2)
C2—C3	1.371 (3)	C2A—C3A	1.380 (3)
C2—C6	1.454 (3)	C2A—C6A	1.460 (3)
O3—C6	1.335 (2)	O3A—C6A	1.325 (2)
O3—C15	1.450 (2)	O3A—C15A	1.451 (2)
C3—C4	1.409 (3)	C3A—C4A	1.411 (3)
C4—C5	1.355 (3)	C4A—C5A	1.354 (3)
C4—H4	0.947 (16)	C4A—H4A	0.905 (18)
C5—H5	0.951 (17)	C5A—H5A	0.988 (17)
C7—H7A	0.9600	C7A—H7AA	0.9600
C7—H7B	0.9600	C7A—H7AB	0.9600
C7—H7C	0.9600	C7A—H7AC	0.9600
C8—H8A	0.9600	C8A—H8AA	0.9600
C8—H8B	0.9600	C8A—H8AB	0.9600
C8—H8C	0.9600	C8A—H8AC	0.9600
C9—C10	1.374 (3)	C9A—C10A	1.386 (3)
C9—C14	1.378 (3)	C9A—C14A	1.386 (3)
C10—C11	1.382 (3)	C10A—C11A	1.370 (2)
C10—H10A	0.9300	C10A—H10B	0.9300
C11—C12	1.367 (3)	C11A—C12A	1.365 (3)
C11—H11A	0.9300	C11A—H11B	0.9300
C12—C13	1.367 (3)	C12A—C13A	1.367 (3)
C13—C14	1.375 (3)	C13A—C14A	1.375 (3)
C13—H13A	0.9300	C13A—H13B	0.9300
C14—H14A	0.9300	C14A—H14B	0.9300
C15—C16	1.427 (3)	C15A—C16A	1.385 (3)
C15—H15A	0.9700	C15A—H15C	0.9700
C15—H15B	0.9700	C15A—H15D	0.9700
C16—H16A	0.9600	C16A—H16D	0.9600
C16—H16B	0.9600	C16A—H16E	0.9600
C16—H16C	0.9600	C16A—H16F	0.9600
			
C3—O1—N1	109.61 (15)	C3A—O1A—N1A	109.90 (14)
N1—C1—C2	111.4 (2)	C1A—N1A—O1A	105.11 (15)
N1—C1—C9	117.5 (2)	N1A—C1A—C2A	111.79 (19)
C2—C1—C9	131.1 (2)	N1A—C1A—C9A	117.62 (19)
C1—N1—O1	105.39 (16)	C2A—C1A—C9A	130.57 (19)
C5—N2—C8	121.6 (2)	C5A—N2A—C7A	121.99 (19)
C5—N2—C7	120.5 (2)	C5A—N2A—C8A	120.80 (19)
C8—N2—C7	117.5 (2)	C7A—N2A—C8A	117.21 (18)
C3—C2—C1	105.32 (19)	C3A—C2A—C1A	105.39 (18)
C3—C2—C6	123.7 (2)	C3A—C2A—C6A	128.1 (2)
C1—C2—C6	130.9 (2)	C1A—C2A—C6A	126.3 (2)
C6—O3—C15	115.92 (18)	C6A—O3A—C15A	117.49 (17)
O1—C3—C2	108.2 (2)	O1A—C3A—C2A	107.78 (18)
O1—C3—C4	118.6 (2)	O1A—C3A—C4A	117.8 (2)
C2—C3—C4	133.1 (2)	C2A—C3A—C4A	134.4 (2)
C5—C4—C3	123.1 (2)	C5A—C4A—C3A	122.7 (2)
C5—C4—H4	122.4 (11)	C5A—C4A—H4A	123.9 (12)
C3—C4—H4	114.5 (11)	C3A—C4A—H4A	113.3 (12)
N2—C5—C4	127.8 (2)	N2A—C5A—C4A	126.5 (2)
N2—C5—H5	112.9 (11)	N2A—C5A—H5A	116.7 (10)
C4—C5—H5	119.3 (10)	C4A—C5A—H5A	116.8 (10)
O2—C6—O3	122.6 (2)	O2A—C6A—O3A	123.2 (2)
O2—C6—C2	125.0 (2)	O2A—C6A—C2A	123.8 (2)
O3—C6—C2	112.4 (2)	O3A—C6A—C2A	113.0 (2)
N2—C7—H7A	109.5	N2A—C7A—H7AA	109.5
N2—C7—H7B	109.5	N2A—C7A—H7AB	109.5
H7A—C7—H7B	109.5	H7AA—C7A—H7AB	109.5
N2—C7—H7C	109.5	N2A—C7A—H7AC	109.5
H7A—C7—H7C	109.5	H7AA—C7A—H7AC	109.5
H7B—C7—H7C	109.5	H7AB—C7A—H7AC	109.5
N2—C8—H8A	109.5	N2A—C8A—H8AA	109.5
N2—C8—H8B	109.5	N2A—C8A—H8AB	109.5
H8A—C8—H8B	109.5	H8AA—C8A—H8AB	109.5
N2—C8—H8C	109.5	N2A—C8A—H8AC	109.5
H8A—C8—H8C	109.5	H8AA—C8A—H8AC	109.5
H8B—C8—H8C	109.5	H8AB—C8A—H8AC	109.5
C10—C9—C14	118.1 (2)	C10A—C9A—C14A	117.9 (2)
C10—C9—C1	121.1 (2)	C10A—C9A—C1A	120.9 (2)
C14—C9—C1	120.8 (2)	C14A—C9A—C1A	121.2 (2)
C9—C10—C11	121.2 (2)	C11A—C10A—C9A	121.7 (2)
C9—C10—H10A	119.4	C11A—C10A—H10B	119.1
C11—C10—H10A	119.4	C9A—C10A—H10B	119.1
C12—C11—C10	119.3 (2)	C12A—C11A—C10A	119.1 (2)
C12—C11—H11A	120.3	C12A—C11A—H11B	120.4
C10—C11—H11A	120.3	C10A—C11A—H11B	120.4
C13—C12—C11	120.5 (2)	C11A—C12A—C13A	120.6 (2)
C13—C12—Cl1	119.8 (2)	C11A—C12A—Cl1A	119.8 (2)
C11—C12—Cl1	119.6 (2)	C13A—C12A—Cl1A	119.6 (2)
C12—C13—C14	119.6 (2)	C12A—C13A—C14A	120.3 (2)
C12—C13—H13A	120.2	C12A—C13A—H13B	119.9
C14—C13—H13A	120.2	C14A—C13A—H13B	119.9
C13—C14—C9	121.2 (2)	C13A—C14A—C9A	120.3 (2)
C13—C14—H14A	119.4	C13A—C14A—H14B	119.8
C9—C14—H14A	119.4	C9A—C14A—H14B	119.8
C16—C15—O3	110.5 (2)	C16A—C15A—O3A	110.5 (2)
C16—C15—H15A	109.5	C16A—C15A—H15C	109.5
O3—C15—H15A	109.5	O3A—C15A—H15C	109.5
C16—C15—H15B	109.5	C16A—C15A—H15D	109.5
O3—C15—H15B	109.5	O3A—C15A—H15D	109.5
H15A—C15—H15B	108.1	H15C—C15A—H15D	108.1
C15—C16—H16A	109.5	C15A—C16A—H16D	109.5
C15—C16—H16B	109.5	C15A—C16A—H16E	109.5
H16A—C16—H16B	109.5	H16D—C16A—H16E	109.5
C15—C16—H16C	109.5	C15A—C16A—H16F	109.5
H16A—C16—H16C	109.5	H16D—C16A—H16F	109.5
H16B—C16—H16C	109.5	H16E—C16A—H16F	109.5

### Hydrogen-bond geometry (Å, º)

**Table:**

*D*—H···*A*	*D*—H	H···*A*	*D*···*A*	*D*—H···*A*
C4—H4···O2	0.95 (2)	2.425 (18)	3.025 (3)	121.1 (13)
C4*A*—H4*A*···O3*A*	0.91 (2)	2.409 (19)	2.996 (3)	122.7 (14)
C16*A*—H16*F*···Cl1*A*^i^	0.96	2.74	3.607 (3)	151

Symmetry code: (i) *x*, *y*, *z*−1.
