# Supplementary figures and images for: Crystal structure of ethyl 3-(4-chloro­phen­yl)-5-[(E)-2-(di­methyl­amino)­ethen­yl]-1,2-oxazole-4-carboxyl­ate
Source: Acta Crystallogr E Crystallogr Commun. 2015 Dec 9;71(Pt 12):o1028. doi: 10.1107/S2056989015023257 (PMC4719961; doi:10.1107/S2056989015023257)

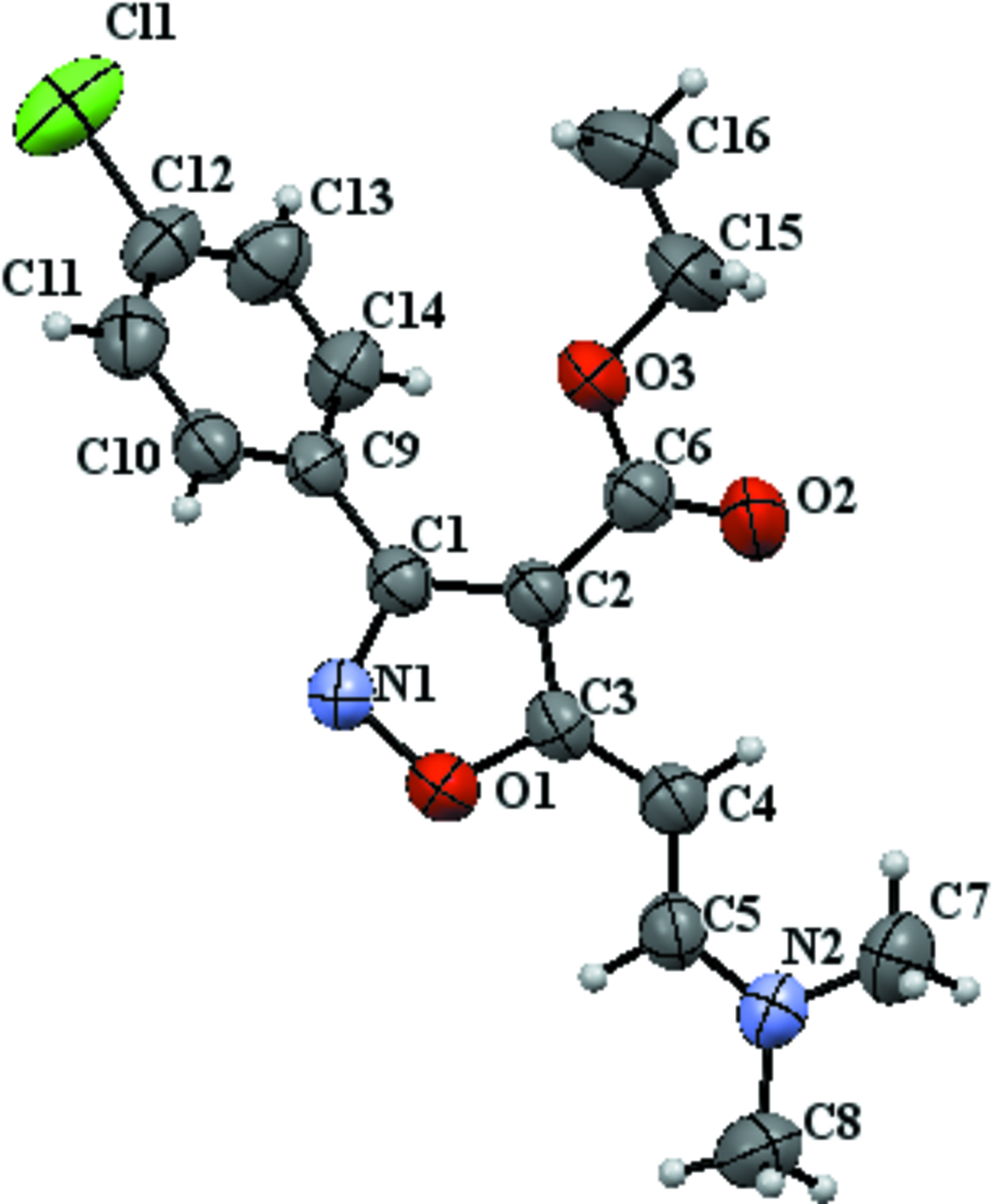

Supplement: Supplementary file 6 [file e-71-o1028-fig1.tif]
